# Cardiac biophysical detailed synergetic modality rendering and visible correlation

**DOI:** 10.3389/fphys.2023.1086154

**Published:** 2023-04-07

**Authors:** Fei Yang, Xiaoxi Wei, Bo Chen, Chenxi Li, Dong Li, Shugang Zhang, Weigang Lu, Lei Zhang

**Affiliations:** ^1^ School of Mechanical, Electrical and Information Engineering, Shandong University, Weihai, China; ^2^ School of Computer Science and Technology, Shandong University, Qingdao, China; ^3^ Pizhou Power Supply Branch of State Grid Jiangsu Electric Power Co., Ltd., Pizhou, China; ^4^ College of Computer Science and Technology, Ocean University of China, Qingdao, China; ^5^ Department of Educational Technology, Ocean University of China, Qingdao, China; ^6^ Department of Radiology, University of Pittsburgh, Pittsburgh, PA, United States

**Keywords:** cardiac synergetic configuration, biophysical detail, WebGL-based rendering, interactive configuration histogram, physical and electrophysiological correlation

## Abstract

The heart is a vital organ in the human body. Research and treatment for the heart have made remarkable progress, and the functional mechanisms of the heart have been simulated and rendered through the construction of relevant models. The current methods for rendering cardiac functional mechanisms only consider one type of modality, which means they cannot show how different types of modality, such as physical and physiological, work together. To realistically represent the three-dimensional synergetic biological modality of the heart, this paper proposes a WebGL-based cardiac synergetic modality rendering framework to visualize the cardiac physical volume data and present synergetic correspondence rendering of the cardiac electrophysiological modality. By constructing the biological detailed interactive histogram, users can implement local details rendering for the heart, which could reveal the cardiac biology details more clearly. We also present cardiac physical-physiological correlation visualization to explore cardiac biological association characteristics. Experimental results show that the proposed framework can provide favorable cardiac biological detailed synergetic modality rendering results in terms of both effectiveness and efficiency. Compared with existing methods, the framework can facilitate the study of the internal mechanism of the heart and subsequently deduce the process of initiation, development, and transformation from a healthy heart to an ill one, and thereby improve the diagnosis and treatment of cardiac disorders.

## 1 Introduction

Globally, the number of people by heart disease is increasing. Heart disease has become a serious threat to human health, ranking among the three leading causes of death. To prevent and cure heart disease, it is necessary to understand the mechanisms underlying cardiac physiology and pathology in depth. Although clinical diagnosis and relevant data have provided significant support for the study of heart disease, it is still challenging to explore physiological mechanism and pathogenesis of the heart to assist in the treatment of heart disease. Due to the restricted experimental environment or condition, the study of the heart is significantly hindered. Therefore, it is crucial to create a virtual heart that can simulate the cardiac function (Zhang et al., 2000).

To help researchers understand the physiological mechanisms of the heart and the etiology of heart disease, computational cardiology models and simulate the heart by comprehensively leveraging cardiac physiology, mathematical modeling methods, and virtual reality (Funk et al., 2008). In 1960, Noble implemented the first computational model of cardiomyocytes for the first time, which opens up the modeling research of cardiac electrophysiological activity (Noble, 1960). So far, researchers have built hundreds of models of various species and types, from subcellular and cellular to tissue and organ levels. Computation and visualization of cardiac models compute and simulate features under physiological and varied pathological states, such as cardiac structure, biomechanics, biochemical, and electrophysiological activity and turn them into graphics and images that replicate the activity processes of the human heart in terms of morphology, structure, and function.

Researchers have built heart models based on geometry (Kerckhoffs et al., 2003; Sermesant et al., 2006), tissue slices (Vetter and McCulloch, 1998; Nielsen et al., 1991; Primoz et al., 2007; Zhang et al., 2016), and imaging data (Virag et al., 2002; Helm Patrick et al., 2005; Viatcheslav et al., 2011; Aslanidi Oleg et al., 2013) to performance the structure of tissues and organs of the heart for the non-invasive research of cardiac function mechanisms. Burton Rebecca et al. (2006). (Gernot et al., 2009) built a high-resolution dual-chamber model of the heart based on ultra-high resolution *ex-vivo* MRI data of the small mammalian heart. The model can show tissue-level details of the cardiac structure. To reveal detailed structures of the human heart, considerable studies have focused on visualizing the cardiac volume data by various algorithms based on direct volume rendering (Liu et al., 2014; Zhang et al., 2011; Wang et al., 2011; Gai et al., 2011; Zhang et al., 2016). In addition, *ex-vivo* MRI images (Vadakkumpadan et al., 2008; Bordas et al., 2011) and micro-CT scans (Stephenson Robert et al., 2012) have been used to reconstruct the entire cardiac conduction system (CSS) semi-automatically.

Because of the complicated cardiac anatomy, some heart tissues cannot be easily distinguished from adjacent tissues when viewed from a particular viewpoint. To improve visualization effects, Zhang et al. (2014a) proposed a method of light enhancement to emphasize specific cardiac tissues while weaken the display of other tissues. However, this method considers the visualization of myocardial fibers orientation and the electrochemical reaction to stimulation conduction. Chen et al. transformed the reconstructed fiber bundles into scalar field that represent their structures based on DTI (Diffusion Tensor Imaging), and then proposed texture synthesis method to synthesize the constructed guidance vector field and sample texture into volume texture. Finally, they established a line-based volume illumination formulation to solve the problem of visualizing myocardial fibers and implemented a GPU-based technique for biological tissue fibers visualizations (Chen et al., 2009a; Chen et al., 2009b; Ming-Yuen et al., 2009). Yuan and Wang, 2014 applied DTMRI (Diffusion Tensor Magnetic Resonance) to analyze myocardial fiber orientation (Yuan et al., 2011) and proposed a mixed filter of the 3D Gauss and directional distance filter that preserves vector directions of myocardial fibers while suppressing noises in vector fields (Yuan and Wang, 2014). On this basis, Yuan tracked the orientation of myocardial fibers and combined cardiac features of scalar and vector to visualize myocardial fiber orientation and the structure of cardiac biological tissues.

In the field of computational visualization of cardiac function, (Edward et al., 2009) built an image-based 3D ventricular model of an infarcted canine heart, which simulates the mechanism of epicardial re-entry morphology. Sato et al. (2009) and Dressler, (2015) simulated the electrical activity of cardiac tissues and organs. However, the model they proposed could not represent the functional and structural characteristics of a real human heart since it is an animal heart model. Burton Brett et al. (2013) of Utah University highlighted the simulated cardiac ischemic regions by non-deterministic visualization. Aslanidi et al. (2011) from the University of Manchester built a complete human atrial model to visualize the multi-scale dynamic behavior of the human atria during the normal rhythm and atrial fibrillation, thus revealing the conduction mechanisms of the electrophysiology of atrial tissue in the normal and arrhythmic conditions. Lu et al. (2015a) built a model of human ventricular ischemia and visually analyzed the effect of acute global ischemia on ventricular rhythm and subsequently on re-entrant arrhythmogenesis (Lu et al., 2015b). Trayanova et al. (2010) studied the mechanism of ventricular arrhythmias by building 3D computational simulation models. Xiong et al. (2017) visualized the cardiac anatomical structure and its physiological functions by CT and computer simulation. Zhang et al. (2012) developed the multi-modality visualization methods for both heart anatomical data and electrophysiological data (Zhang et al., 2012; Zhang et al., 2016; Zhang et al., 2014b). Vahid et al. (2014) applied the three-dimensional bionic technique to construct models to analyze the structure and function of the failing heart. These methods offer effective observation method representing the anatomical and biophysical information in particular regions of interest of the heart under both normal and pathological conditions.

Direct volume rendering generates two-dimensional images based on three-dimensional data fields. Using a user defined transfer function, it composes a result image by aggregating the colors and opacities of relevant voxels of the volumetric data sets (Kruger and Westermann, 2003). Among them, Volume Ray Casting (Ljung et al., 2016) is a common technique for volume visualization which displays the salient characteristics of the volume set. Although it is not photo realistic, it shows important characteristics of the dataset. Due to its capability of directly displaying obscured internal features and demonstrating more information about the volume data, direct volume rendering has drawn increasing attention in the research of cardiac computation and visualization. Current cardiac rendering methods focus on the single modality, so these methods cannot demonstrate the synergistic associations between physical and physiological modalities.

In this paper, we construct a web framework based on WebGL for the visualization of the heart, implement the visual computation of cardiac modality and its coordinated functions, and provide a realistic representation of the 3D information of organic functional modalities, such as cardiac structure, biochemical reactions, and electrobiological activities from a holistic perspective. Meanwhile this framework enables direct web low-level 3D graphics acceleration which significantly improves web rendering speed, and the space can be saved compared to traditional visualization systems. And owing to the advantage of cross-platform of WebGL, our framework is convenient for porting and thus has superb flexibility. The main contributions of the paper are as following.

1. First we innovatively propose a WebGL-based rendering framework for real-time network visualization of both the complete physical modality and real physiological functions.
2. We construct the interactive cardiac physical modality histogram and achieve the local details rendering. The physical structure and specific tissues of the heart are realistically and interactively demonstrated.
3. We further present a novel cardiac physical-physiological correlation visualization method by constructing the correlation module to help observe synergistic associations between physical-physiological modalities for deep understanding of the nature of cardiac physical-physiological functions.

The remainder of the paper is organized as follows. Section 2 introduces cardiac biophysical modality volume data and the implementation of the cardiac synergetic modality rendering, including the interactive cardiac modality histogram based local detail rendering. In Section 3, the cardiac biological correlation module is constructed and the visualization of cardiac physical-physiological correlation is presented. In the last part, the conclusion of this study is proposed.

## 2 Cardiac synergetic modality rendering

Visualization is the process of transmitting and expressing information through graphical representation. Scientific visualization, including surface rendering and volume rendering, can extract complex information from 3D volume data and represent 3D phenomena through graphics, thus transferring and expressing information effectively. Volume rendering displays three-dimensional data field as a two-dimensional image, thereby not only the shape, boundary and surface information are depicted, but also the internal hidden information can be revealed. This work achieves cardiac synergetic biophysical modality rendering based on the WebGL ray casting volume rendering model, offering the user different levels of the biological characteristics of the heart.

### 2.1 Cardiac biophysical modality volume data

In the field of volume rendering, the three-dimensional data field is a structured dataset consisting of three-dimensional grids, which is composed of a finite number of uniformly distributed voxels. Cardiac synergetic biophysical modality rendering in this paper works with 3D heart volume data which are the regular samples of scalar (*f*: *R* ^3^ → *R*) fields. The volume data includes the biological structure volume from the Visible Human Project and the resultant computational electrophysiology volume.

### 2.2 WebGL based rendering framework

In this paper we build a WebGL based framework of cardiac biological cooperative construction volume rendering. Due to the fact that 3D texture is not supported in WebGL, the volume data stored in the raw file is thus parsed, then layered into a large 2D texture, and finally the volume is rendered using 3D texture sampling.

When obtaining the dimension of volume data, we thus determine 2D mapping layout scheme as well as the range of 2D texture sizes which satisfies Eq. 1:

$$\begin{array}{c} W2*H2\geq W1*H1*L1 \end{array}$$ (1)

Here *W*1 is the width of volume data and *H*1 is the thickness of volume data. *L*1 demonstrates the length of volume data. *W*2 is the width of 2D mapping and *H*2 represents the length of the 2D mapping. We then allocate space four times the texture size for values of RGBA and initialize the texture data. R, G and B represent red, green and blue colors respectively, and A represents opacity. After the data is stored in the 2D texture, 3D texture sampling is performed for rendering.

Ray casting algorithm is the most straightforward volume rendering method that can generate high quality images. Given the viewpoint, we firstly calculate the direction of the rays as in Eq. 2 when the pixels on the screen are selected:

$$\begin{array}{c} vec3Dir=norm\left(Px*Vx+Py*Vy+2.0*Vz\right) \end{array}$$ (2)

where vec3Dir is the direction of the ray. *Px* and *Py* are the *x* coordinate and *y* coordinate of the pixel on the screen which the ray passes through. *Vx*, *Vy* and *Vz* are the *x*-axis, *y*-axis and *z*-axis respectively of View-coordinate.

The intersection sample voxels then arise along the ray direction while the ray passes through the volume data. Assume that previous sample voxel has been acquired, the location of current voxel on the ray can be determined according to the step size which is the distance to move within the volume data along the view ray. The opacity and color of current voxel can then be calculated as:

$$\begin{array}{c} A_{s}=\frac{w_{O}*P_{v}}{P_{s}} \end{array}$$ (3)

$$\begin{array}{c} S_{s}={w_{L}*C}_{v}*A_{s} \end{array}$$ (4)

where *P*_*v*_ is the scalar value of the voxel *v* in the volume, and *C*_*v*_ is the color obtained through the designed transfer function based on *P*_*v*_. *P*_*s*_ is the scalar value of the sampled voxel *s* on the ray. *A*_*s*_ is the opacity of *s* and *C*_s_ is the color of *s*. w_*L*_, w_*O*_ are the general weights for light and opacityrespectively. The final color $C_{p}^{k}$ of pixels on the screen corresponding to the ray is subsequently accumulated as in Eqs 5, 6:

$$\begin{array}{c} A_{p}^{k}=A_{p}^{k-1}+A_{s} \end{array}$$ (5)

$$\begin{array}{c} C_{p}^{k}=C_{p}^{k-1}+\left(1-A_{p}^{k-1}\right)*S_{s} \end{array}$$ (6)

Here $A_{p}^{k}$ is the opacity of the pixel. Once all the sample voxels on the ray have been processed, or the accumulated result reaches the threshold value, the calculation of color and opacity for sample voxels on the ray intersecting with the volume is completed. And the resulting rendered image can be generated. Supplementary Algorithm S1.

### 2.3 Interactive modality histogram based cardiac detail rendering

Based on the ray casting, the entire cardiac biological structure can be rendered. However, in order to more effectively aid researchers in exploring the internal modality details of the heart, a more precise representation of the heart is necessary.

#### 2.3.1 Transfer function

So far, researchers have conducted numerous studies aiming to improve the speed and quality of volume rendering. The critical factor affecting these two important indices can be traced back to the design of the transfer function. Transfer function transforms the values of sample voxels in the volume data into optical properties that are visible to human eyes, such as color, opacity, etc. This allows for the exploration of the internal structure of various objects in the resulting rendered image. The transfer function can be formally defined as:

$$\begin{array}{c} T:x|\to \left\{c,a\right\},x\in R_{n} \end{array}$$ (7)

In Eq. 7, $\left\{c,\alpha \right\}$ is usually a two-tuple consisting of color and opacity. x is the attribute value of the sample voxels in volume data. The dimension n of x is the number of attributes. The space defined by these attributes is referred to as the feature space. In this paper the transfer function is designed through the constructed interactive cardiac modality histogram, so as to achieve local detail rendering of cardiac volume data.

#### 2.3.2 Interactive cardiac modality histogram

We first count the number of myocyte voxels of different tissue of the cardiac physical modality in the volume data, and then construct the interactive cardiac modality histogram based on the statistics result. In the histogram, the value of tissues in the volume data increases from left to right, and the number of relevant voxels is expressed in the form of a vertical bar. The higher the bar, the more myocytes of the tissue are present, indicating a larger volume of the tissue in the heart.

**FIGURE 1:**
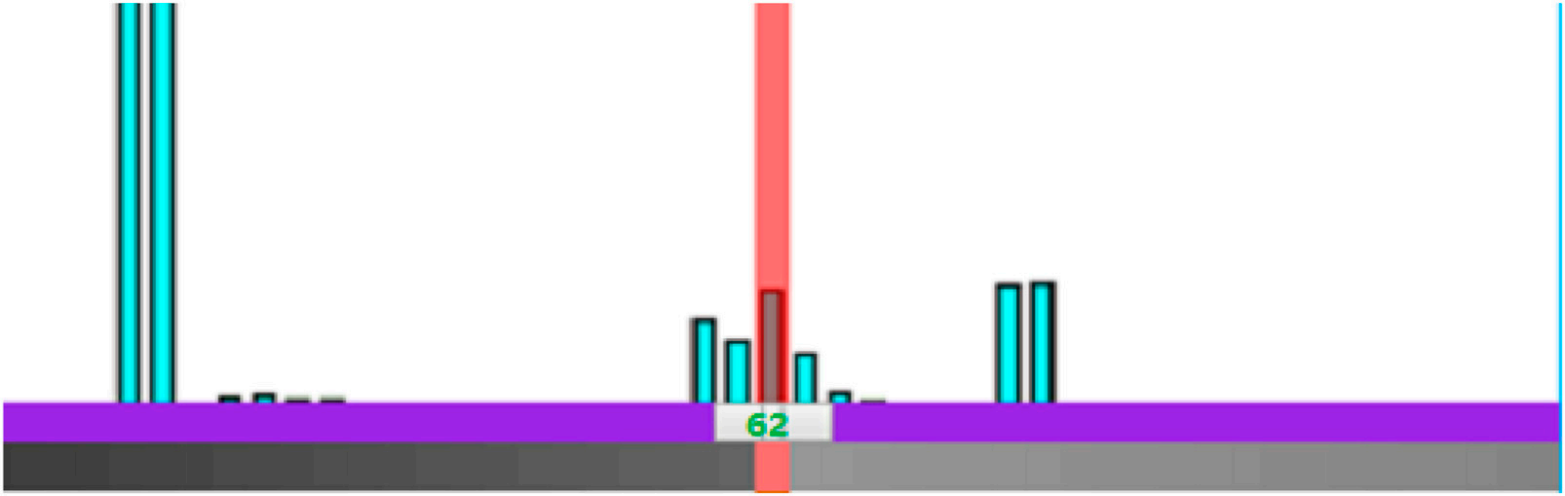
The interactive cardiac physical modality histogram.

Although the cardiac modality histogram clearly shows the statistical characteristics of cardiac tissue, it lacks interactivity, making it inconvenient for users. By leveraging WebGL we add control points to the histogram according to the value of a certain cardiac tissue, as shown in Figure 1. Users can thus control local rendering by setting control points on the histogram, resulting in modality histogram based interactive cardiac detailed rendering.

#### 2.3.3 The local detailed modality rendering

**FIGURE 2:**
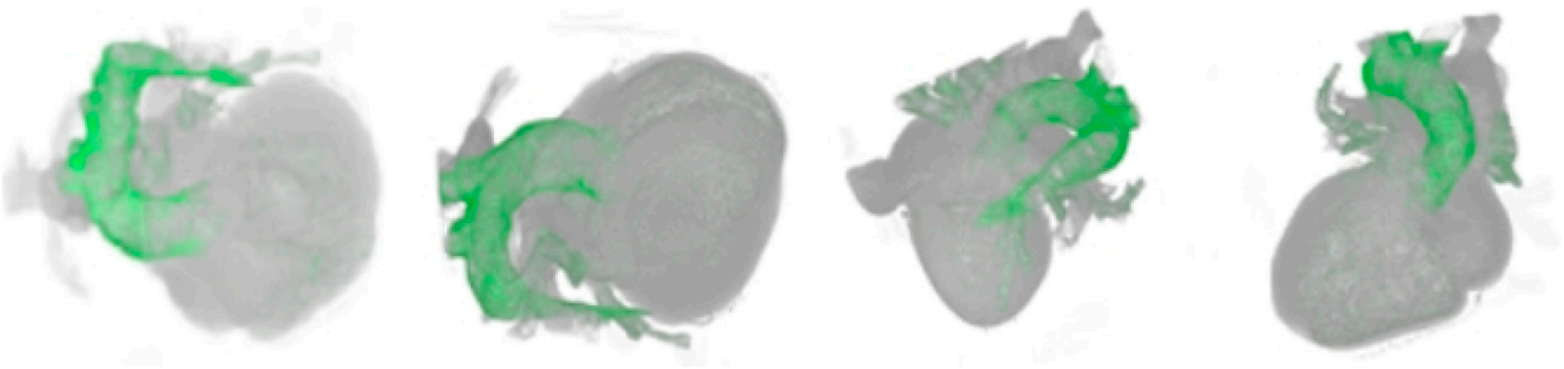
Rendering result of the outer wall of arterial vessels from different viewpoints.

When the control point is added, the color and opacity values of the control point can then be set and be assigned to those corresponding myocyte voxels. The transfer function texture is subsequently recalculated on the basis of the new color and opacity value, and the texture of volume data is regenerated. Eventually the texture map of the transfer function is passed to the shader and applied during rendering. Through the constructed interactive cardiac modality histogram, the biological modality details of the heart can be clearly highlighted and revealed. According to the height of the column with the value of 62 in the histogram in Figure 1, we can conveniently determine the volume proportion of the outer wall of the artery which corresponds to this value in the cardiac volume data. Meanwhile when we add a control point for the column and set its color to green, along with a specified opacity value, the scalar value of the myocyte voxel is mapped to the opacity and color, indicating that the transfer function is implemented. As a result, the outer wall of the artery (green) is highlighted in the final rendering image. The local detail rendering of the arterial outer wall tissues from different viewpoints are shown in Figure 2.

### 2.4 Synergetic electrophysiological rendering

#### 2.4.1 Electrophysiological volume data

Electrophysiological volume data plays an important role in the study of cardiac organs. It reflects the electrical activity of the cardiac tissue at a certain moment. Throughout a complete cycle from depolarization to repolarization of the heart, the action potentials of various cardiac tissues in the electrophysiological volume data are ultimately integrated into the electrocardiogram (ECG). By analyzing the ECG, medical experts can thus diagnose cardiac electrophysiological function and extrapolate dynamic changes of the function over a certain time period.

Similar to cardiac biophysical volume data, the three-dimensional action potential matrix of electrophysiological volume data is also sliced into a group of two-dimensional matrices. Assume that the action potential value of a cardiac tissue cell at a specific moment is *v*, this value will satisfy the following condition:

$$\begin{array}{c} -90\leq v\leq 90,v\in R \end{array}$$ (8)

To facilitate rendering, the action potential value is subsequently linearly mapped to the range from 0 to 255.

#### 2.4.2 Synergetic rendering of cardiac electrophysiological modality

Cardiac electrophysiological rendering demonstrates the three-dimensional action potential in the cardiac tissue at a certain time. Assigned color during electrophysiological rendering is related to the value of action potential. Since the range of action potential value is different from the color value range, conversion is required to obtain the color from the corresponding action potential value. The conversion formula is as follows:

$$\begin{array}{c} c=\left\{\begin{array}{c} \lceil \left.\frac{64}{45}v\right]+128,\left(\frac{64}{45}v-\lfloor \frac{64}{45}v\rfloor \geq \frac{1}{2}\right)\\ \lfloor \frac{64}{45}v\rfloor +128,\left(\frac{64}{45}v-\lfloor \frac{64}{45}v\rfloor \geq \frac{1}{2}\right) \end{array}\right. \end{array}$$ (9)

**FIGURE 3:**
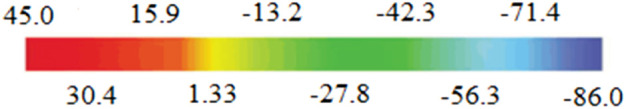
Chromatogram of action potential values.

Where c is the color value. The rendering chromatogram is shown in Figure 3. From Figure 3, we can see that the range of action potential value is from −86 mv to 45 mv, and the corresponding color changes gradually from blue to red.

Different from cardiac biophysical modality rendering, electrophysiological modality rendering requires a distinct rendering method for the shader. In this work, a static rendering scheme is chosen for the shader, based on the correspondence between the action potential value and the color spectrum. The scheme involves building a one-dimensional lookup table that stores the rendering colors for each tissue, as well as another table which stores opacity. Before activating the shader for rendering, our method acquires the corresponding color and opacity from the lookup table according to the action potential values. By modifying the opacity of the tissue voxels in the opacity lookup table, those focused tissues are highlighted in the rendering image owing to the reduction of occlusion by other contextual tissues which is assigned to lower opacity and therefore more transparent.

## 3 Visualization of cardiac physical-physiological correlation

**FIGURE 4:**
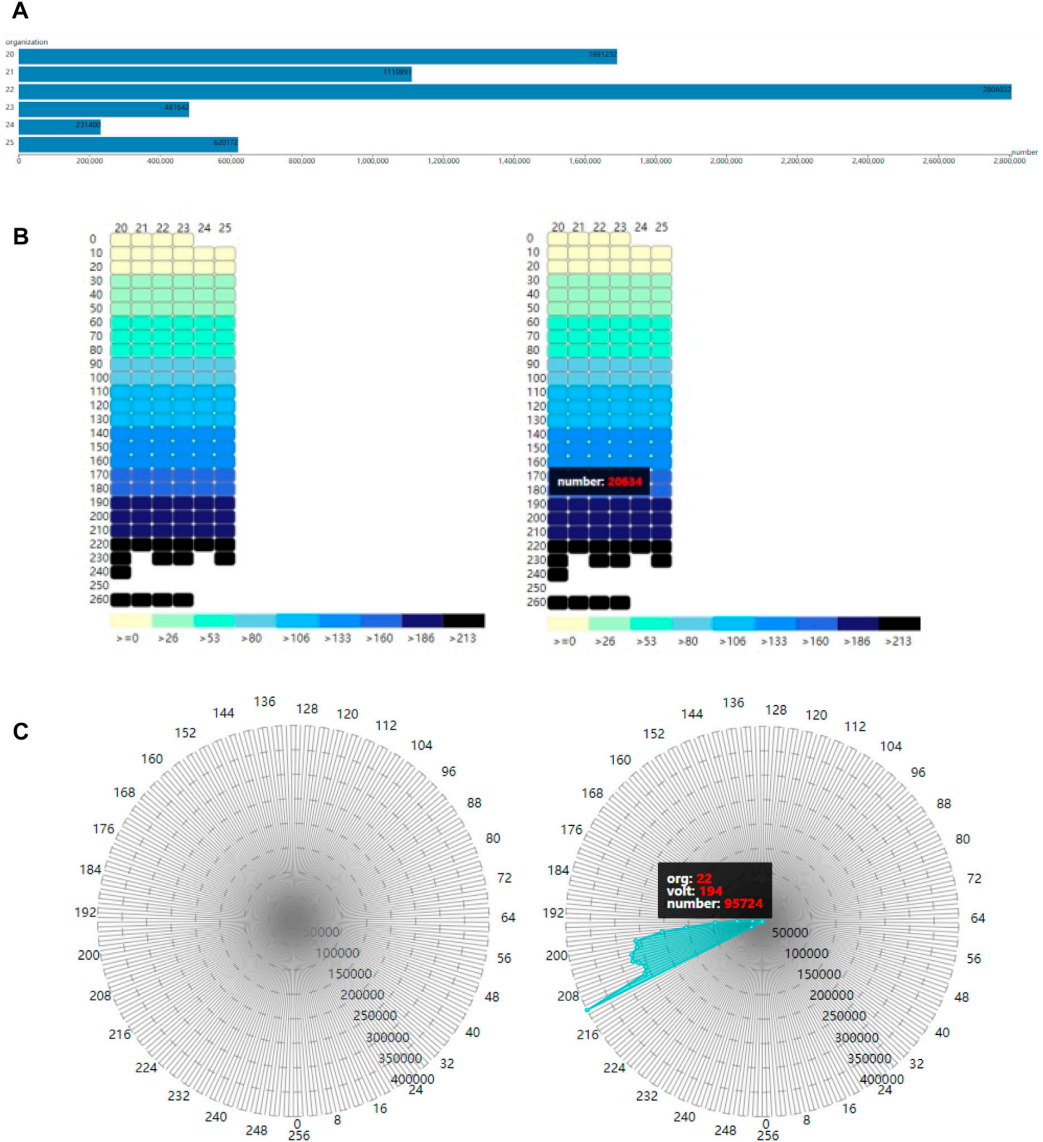
Three modules for cardiac physical and physiological modals **(A)**. Tissue-myocyte module **(B)**. Tissue-electrophysiology module **(C)**. Electrophysiology-myocyte module.

In addition to cardiac synergetic rendering, visualization of physical-physiological correlations is also presented in our proposed framework. This allows researchers or medical experts to analyze both cardiac physical modal and physiological modal information, as well as the relationship between them more directly, providing them a better understanding of cardiac physical and physiological situations. This work builds three modules: tissue-myocyte module, tissue-electrophysiology module and electrophysiology-myocyte module, as shown in Figure 4. The tissue-myocyte module shows the relationship between each cardiac tissue and its constituent myocytes. The tissue-electrophysiology diagram shows the correlation between the cardiac tissue and the action potential at a certain time. And the electrophysiology-myocyte shows the relationship between action potential of the cardiac tissue and its myocytes voxels in the volume data.

### 3.1 Cardiac physical-physiological correlation data

The original cardiac volume data used in this work are two individual volume data of the same heart. One volume data contains the cardiac tissue value of the myocytes, and another contains the action potential value of the same myocytes. In this section, the two-volume data are integrated to store the three-dimensional spatial position of the myocytes, as well as the corresponding tissue value and action potential value.

### 3.2 Construction of module

#### 3.2.1 Tissue-myocyte module

The purpose of the tissue-myocyte module is to visualize physical statistical characteristics of the myocytes of cardiac tissues. The linear scale of this module is determined by the length of the container and the maximum number of myocytes among all the tissues. The number of myocytes in various tissues is then scaled proportionally to the length of the corresponding bar. The constructed tissue-myocyte module is shown in Figure 4A.

#### 3.2.2 Tissue-electrophysiology module

In our framework, the tissue-electrophysiology module displays the two-dimensional elements of cardiac tissue and the associated action potential. The action potential values and tissue values are arranged in rows and columns, respectively, such that the intersection of the rows and columns represents the number of myocytes in a tissue with a specific action potential value. This allows for functional refinement of the tissue-myocyte module. In the system tissue values range from 20 to 25, representing the left ventricle endocardium, myocardium of the left ventricle, epicardium of the left ventricle, the right ventricle endocardium, myocardium of the right ventricle, and epicardium of the right ventricle. Action potential values in these tissues range from 0 to 255 and are divided into 26 segments in the tissue-electrophysiology module.

By utilizing the action potential value and the tissue value, the exact position of each small rectangle in the tissue-electrophysiology diagram can be calculated, and the color of the small rectangle is determined based on the value of the corresponding action potential segment. The color panel for electrophysiological values is then displayed below the tissue-electrophysiology module. The final tissue-electrophysiology module and its associated panel are depicted in the left of Figure 4B. In the right of Figure 4B, when the user selects a tissue value of 20 and the correlated action potential segment of 180 in the tissue-electrophysiology diagram, the number of myocytes with these two values appears on in tissue-electrophysiology module. This indicates that there are 20,634 cells with action potential values ranging from 180 to 189 in the left ventricle endocardium.

#### 3.2.3 Electrophysiology-myocyte module

To further specify the function of the tissue-electrophysiology module, our work has constructed the electrophysiology-myocyte module to exhibit the number of myocytes with various action potentials for a certain cardiac tissue. The required data for this module contains the tissue value, action potential value, the number of myocytes and correspondence between each other among them.

The voxels in the cardiac electrophysiological volume data with and action potential value of −1 represent myocyte voxels without electrophysiological feature. To avoid confusion, these voxels are assigned a value of 256. The improved volume data contains values in the range of 0–256, which provides 257 action potential values for myocytes in various cardiac tissues. The constructed electrophysiology-myocyte module includes eight regular polygons with 257 edges, where each edge represents a specific action potential value. The circumcircles of the polygons have different concentric diameters which demonstrate the number of myocytes of 50,000, 100,000, 150,000, 200,000, 250,000, 300,000, 350,000, 400,000 from inside to outside. The 257 action potential values are displayed as points on the axis of the electrophysiology-myocyte diagram. To improve visual clarity, only points with values that are in multiples of 8 appear on the outermost polygon edges in the diagram. The constructed electrophysiology-myocyte module is shown on the left side of Figure 4C. On the right side of Figure 4C, when a tissue and electrophysiological value are selected, the corresponding number of myocyte voxels is exhibited in the electrophysiology-myocyte diagram. From the presented result, we can see that there are 95,724 myocyte voxels with the action potential value of 194 in the epicardium of the left ventricle which has the value of 22.

The coordinate of each point on the electrophysiology-myocyte diagram is determined through its action potential value and the number of relevant myocyte voxels. First the distance between each point and the center of the electrophysiology-myocyte diagram, which is the radius of the circumcircle where the point is located is calculated as in Eq. 10:

$$\begin{array}{c} r=rc*\left(n-rangeMin\right)/\left(rangeMax-rangeMin\right) \end{array}$$ (10)

where *rc* is the radius of the circumcircle of the outermost regular polygon of the electrophysiology-myocyte diagram, and *n* is the number of myocytes. *RangeMax* is the maximum number of myocytes presented in the outermost polygon, while *rangeMin* is equal to 0.

The position of the corresponding point on the electrophysiology-myocyte diagram can then be obtained using the calculated radius *r* as in the following equation:

$$\begin{array}{c} x=r*\sin\left(ap*onepiece\right) \end{array}$$ (11)

$$\begin{array}{c} y=r*\cos\left(ap*onepiece\right) \end{array}$$ (12)

where *ap* is the action potential value of the point, $onepiece=\frac{2\pi }{257}$ .

### 3.3 Physical-physiological correlation

In our framework, the tissue-myocyte module, tissue-electrophysiology module and electrophysiology-myocyte module are not isolated from each other. Cardiac tissues and their internal electrophysiological characteristics are presented and correlated to each other through the tissue-myocyte module and tissue-electrophysiology module. The further refined electrophysiology-myocyte module shows the distribution details of electrophysiological physical quantities of cardiac tissues. Meanwhile there are control relationships between the three modules. The tissue-myocyte module can control the display of the tissue-electrophysiology module and the electrophysiology-myocyte module, while the tissue-electrophysiology module has the capability of manipulating the demonstration of the electrophysiology-myocyte diagram and thus further refines the visualization, so that the electrophysiology-myocyte module can show the distribution of the myocytes within a specific action potential segment for a certain cardiac tissue. The coordinates of each point on the electrophysiology-myocyte diagram are computed as in Eq. 13, where *vt* is the action potential value and *v* are the value which the user set. When *vt* is equal to *v* or Eq. 13 is satisfied, the computation of the coordinates of the point is the same as Eqs 11, 12. Otherwise, both the *x* coordinate and *y* coordinate of the point are assigned 0.

$$\begin{array}{c} vt+10-vt\%10=v \end{array}$$ (13)

## 4 Result

**FIGURE 5:**
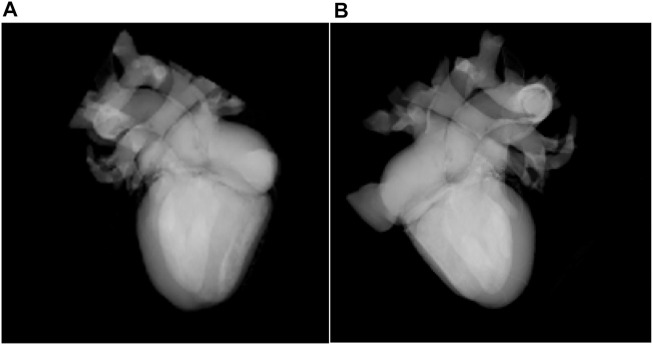
Rendering of the biological structure of the heart **(A)**. Rendering result from one viewpoint **(B)**. Rendering result from a different viewpoint.

The WebGL-based rendering of the biological structure of the heart is shown in Figures 5A, B. In Figure 5A, original rendered cardiac biological structure is demonstrated, and aorta, pulmonary artery, pulmonary vein, superior and inferior vena cava, and cardiac atrium and ventricle are explored. Through WebGL-based interaction rendering, researchers can select the viewpoint by controlling the rendering canvas. Figure 5B shows these tissues from a different viewpoint.

**FIGURE 6:**
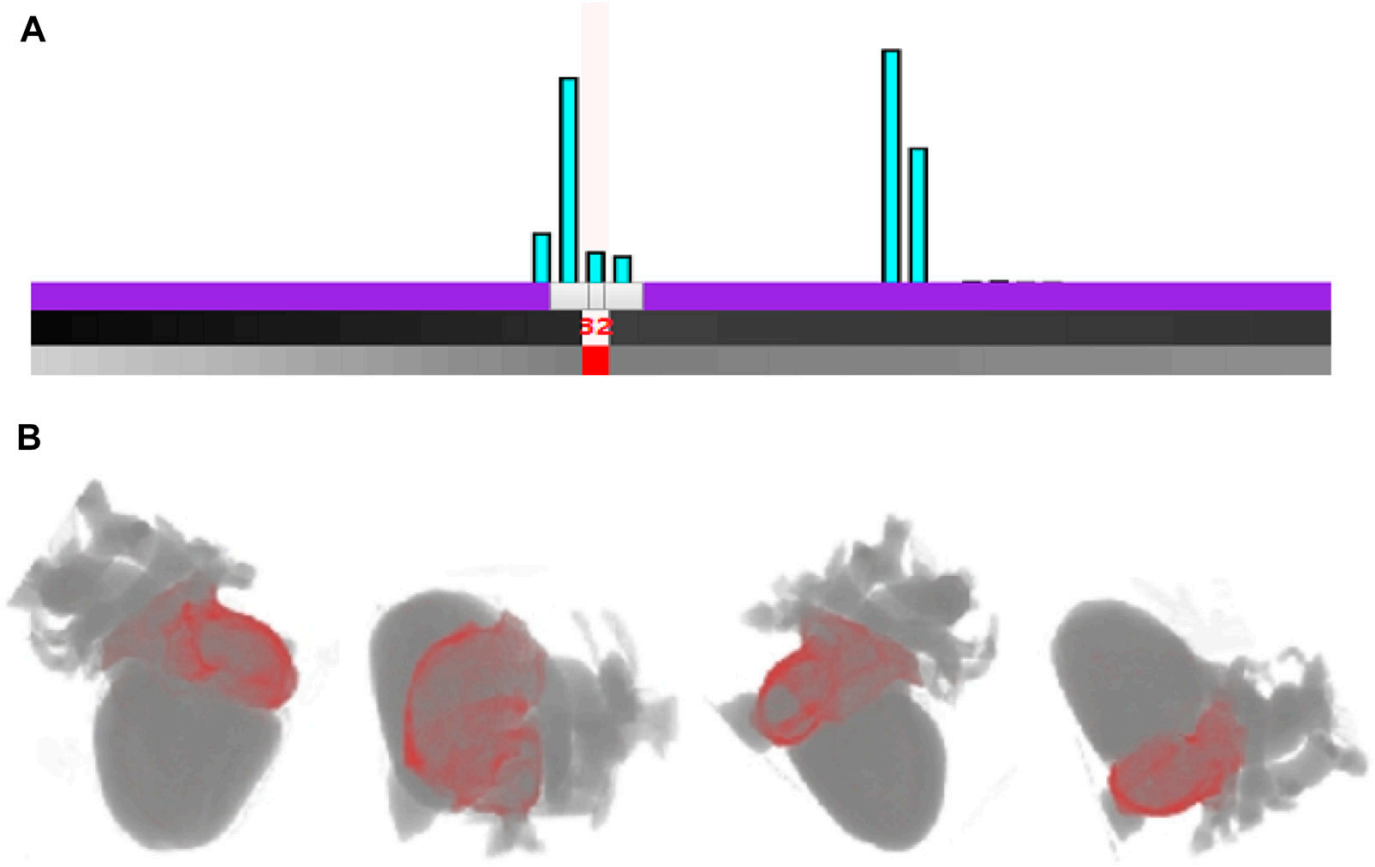
Rendering of the right atrium based on the interactive histogram **(A)**. The interaction with cardiac modality histogram by adding control point and setting the color for the right atrium **(B)**. Rendering result of the right atrium outer wall of arterial vessels from different viewpoints.

Figure 6 shows the interactive histogram-based rendering of the right atrium. In the histogram, the myocyte voxels with tissue value of 32 correspond to the right atrium, as shown in Figure 6A. By controlling the interactive histogram, the exact shape of the right atrium (red) and its position in the heart are presented, as shown in Figure 6B.

**FIGURE 7:**
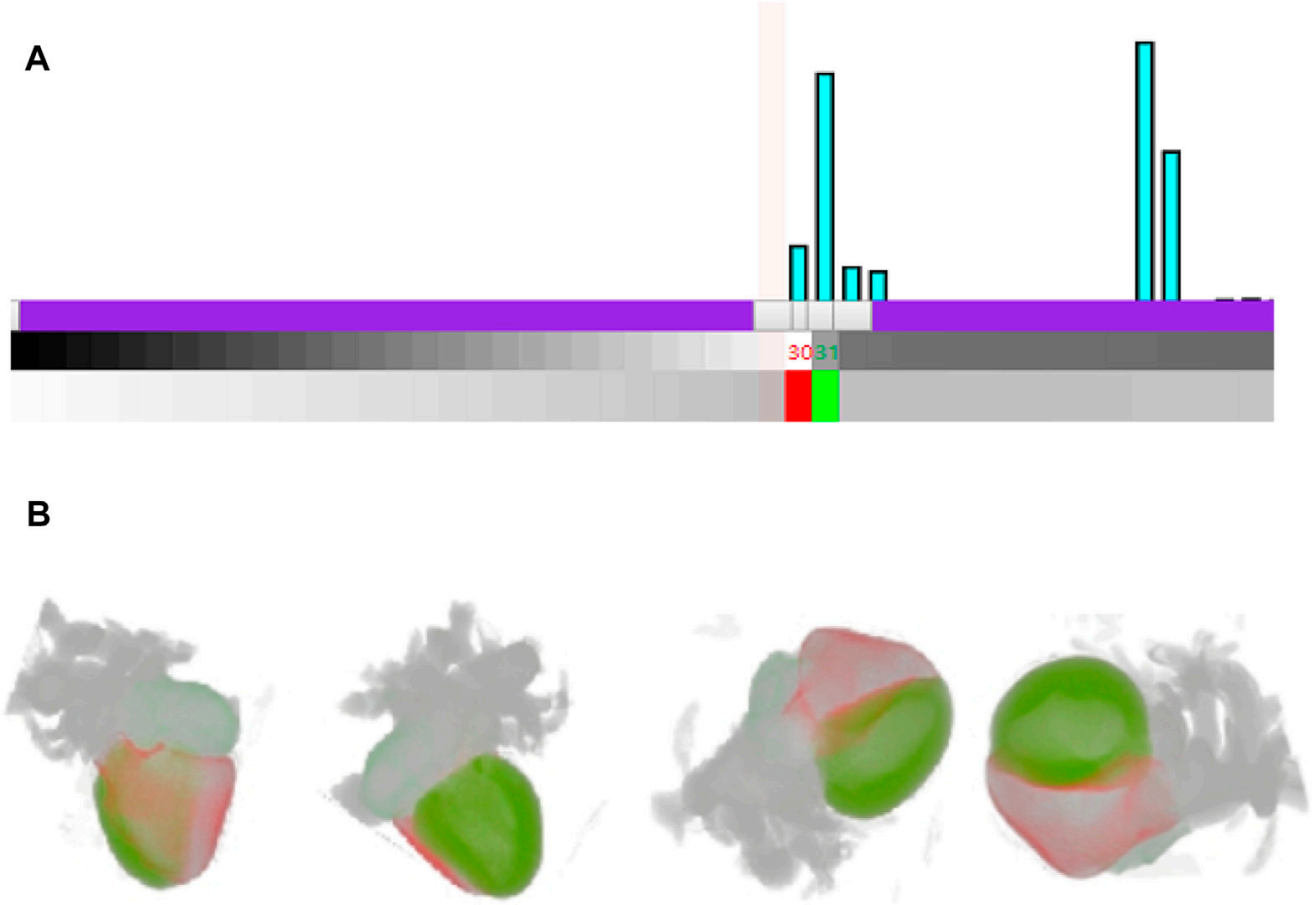
Interactive cardiac multi tissues rendering **(A)**. The interaction with cardiac modality histogram by adding control point and setting the color for the right ventricle and left ventricle **(B)**. Rendering result of the right ventricle and left ventricle from different viewpoints.

Through the interactive histogram, researchers can also interactively control cardiac multi-tissue rendering, as shown in Figure 7. It is obvious in the histogram in Figure 7A that there is a large difference in the number of voxels between the two tissues of right ventricle and left ventricle, with tissue values of 30 and 32 respectively, indicating that the left ventricle is significantly larger than the right ventricle. Researchers can add control points for the relevant voxels of the right ventricle and left ventricle in the interactive histogram, and then set the color and opacity for the two types of voxels through the two control points. Figure 7B highlights the right ventricle (red) and left ventricle (green) from different viewpoints. The shape and size of the two tissues are presented in the rendering result image. Meanwhile the three-dimensional position in the heart as well as the relative spatial position relationship between right ventricle and left ventricle are also distinctly uncovered.

**FIGURE 8:**
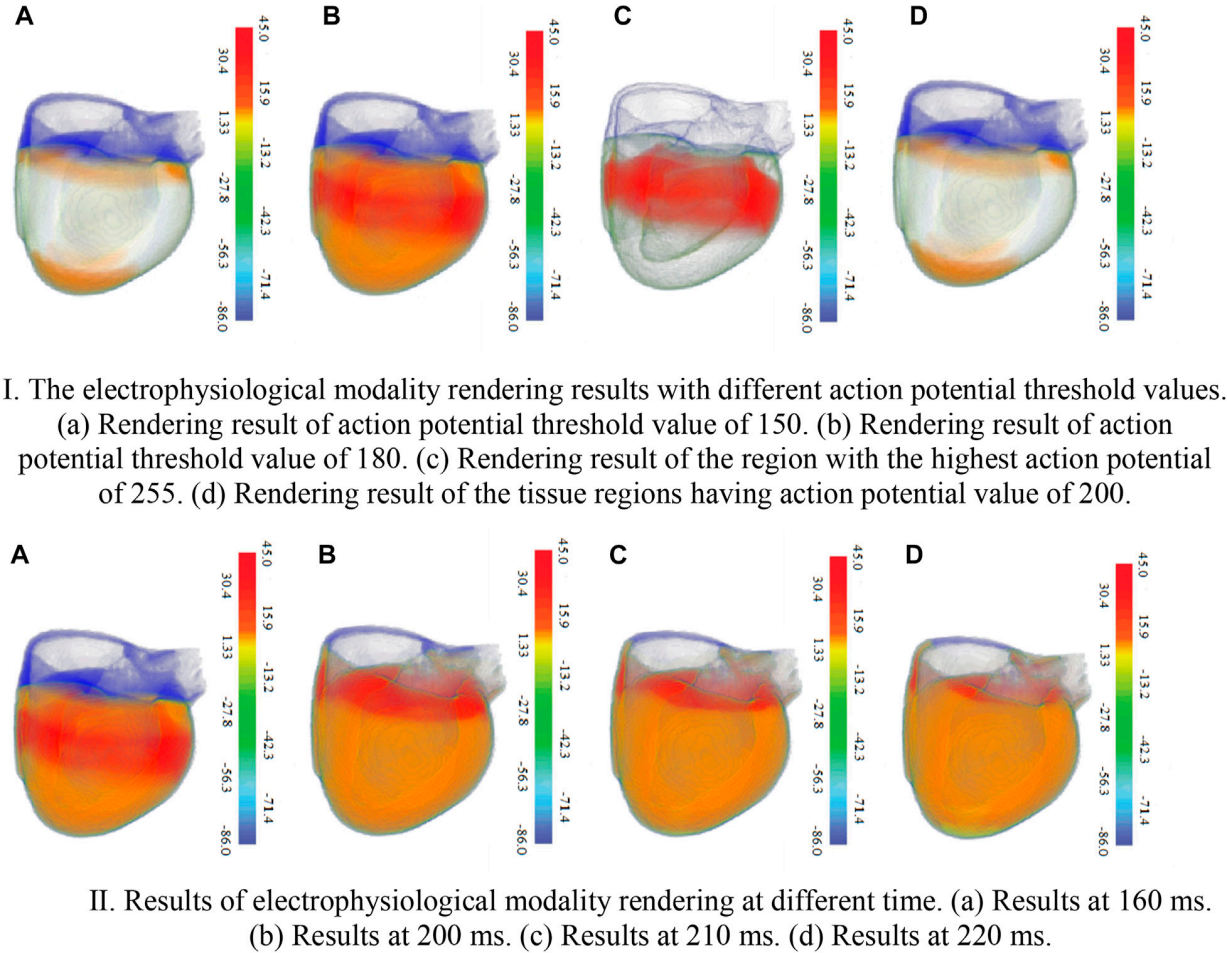
The electrophysiological modality rendering results. Ⅰ. The electrophysiological modality rendering results with different action potential threshold values. 426 (a). Rendering result of action potential threshold value of 150 (b). Rendering result of action 427 potential threshold value of 180 (c). Rendering result of the region with the highest action potential 428 of 255 (d). Rendering result of the tissue regions having action potential value of 200. Ⅱ. Results of electrophysiological modality rendering at different time (a). Results at 160 ms. 432 (b) Results at 200 ms. (c) Results at 210 ms. (d) Results at 220 ms.

Figure 8 shows the electrophysiological modality rendering results with different action potential values and opacities. In Figure 8Ia, since the threshold of maximum action potential value to be demonstrated is predetermined to 150, only myocardial cells of the biventricular tissues with the action potential value below the threshold are rendered, and those with values beyond the threshold are not rendered. In Figure 8Ib, raising the maximum value to 180 results in the rendering of most cells. Figure 8Ic shows the rendering for the regions of the biventricular tissues containing the myocardial cells with the highest action potential value of 255. The rendering result of the tissue regions having an action potential value of 200 is presented in Figure 8Id. Rendering of electrophysiological modality at different times are shown in Figure 8II.

**FIGURE 9:**
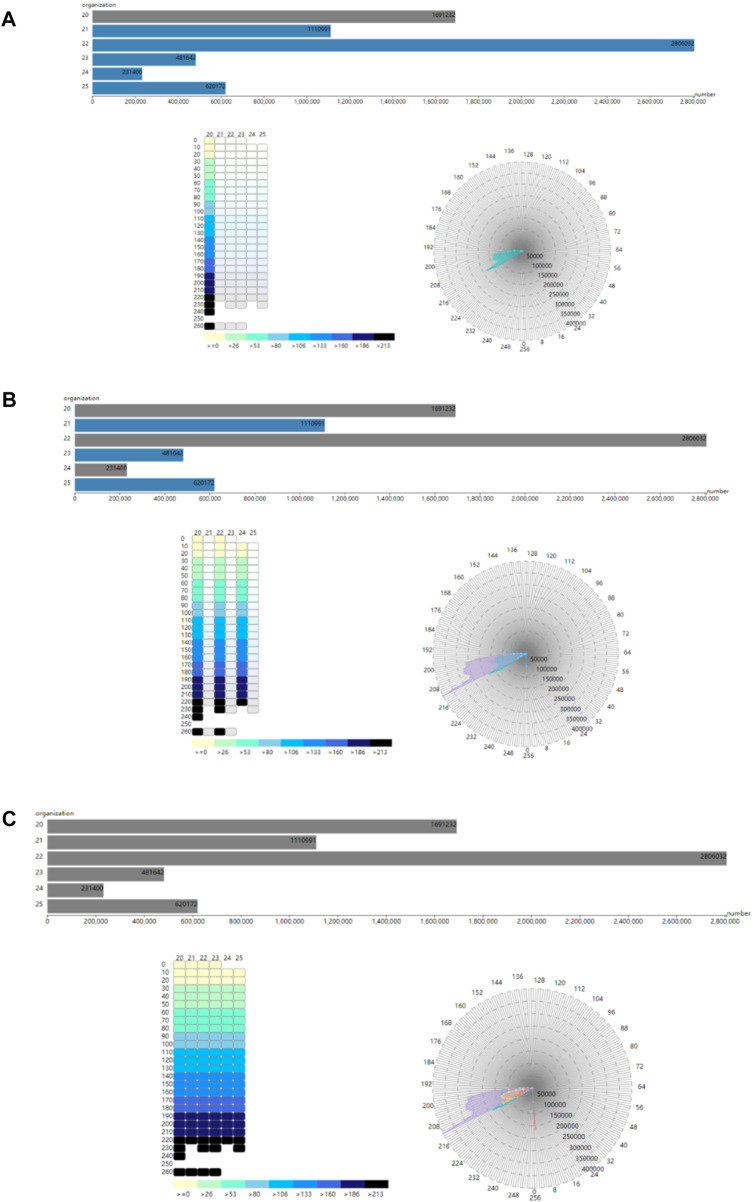
The correlation of the tissue-myocyte module, tissue-electrophysiology module and electrophysiology-myocyte module **(A)**. The association between the three modules when the selected tissue value is 20 **(B)**. The association between the three modules when the selected tissue value is 20, 22, and 24. **(C)** The association between the three modules when the selected tissue value is 20, 21, 22, 23, 24, and 25.

Cardiac physical-physiological correlation between physical and physiological modalities can be visualized based on the tissue-myocyte module, tissue-electrophysiology module and electrophysiology-myocyte module, as shown in Figure 9. In Figure 9A, when only the bar with the cardiac tissue value of 20 representing the left ventricular endocardium is selected, the colour of this bar changes from blue to grey. In the tissue-electrophysiology module, the opacity of rectangles with the tissue value of 20 in a row also changes to 1, while the opacity of the small rectangles in the remaining columns becomes 0.1. Simultaneously, the number of myocytes having the electrophysiological feature in the left ventricular endocardium is demonstrated in the electrophysiology-myocyte module. When selecting the bars representing the left ventricle endocardium, epicardium of the left ventricle, and myocardium of the right ventricle in the tissue-myocyte module, i.e., the bars with the cardiac tissue value of 20, 22, and 24 respectively, their electrophysiological values are all presented in the relevant rectangle columns of the tissue-electrophysiology diagram. In the meantime, the number of myocytes of these three tissues with the action potential highlighted in the tissue-electrophysiology diagram are updated and displayed in the electrophysiology-myocyte diagram, as shown in Figure 9B. In Figure 9C, the endocardium, myocardium, epicardium of the left ventricle and right ventricle are selected and action potential of the cells in them is simultaneously illuminated in the tissue-electrophysiology diagram. The distribution of action potential in the six cardiac tissues are also associatively demonstrated in the electrophysiology-myocyte module.

**FIGURE 10:**
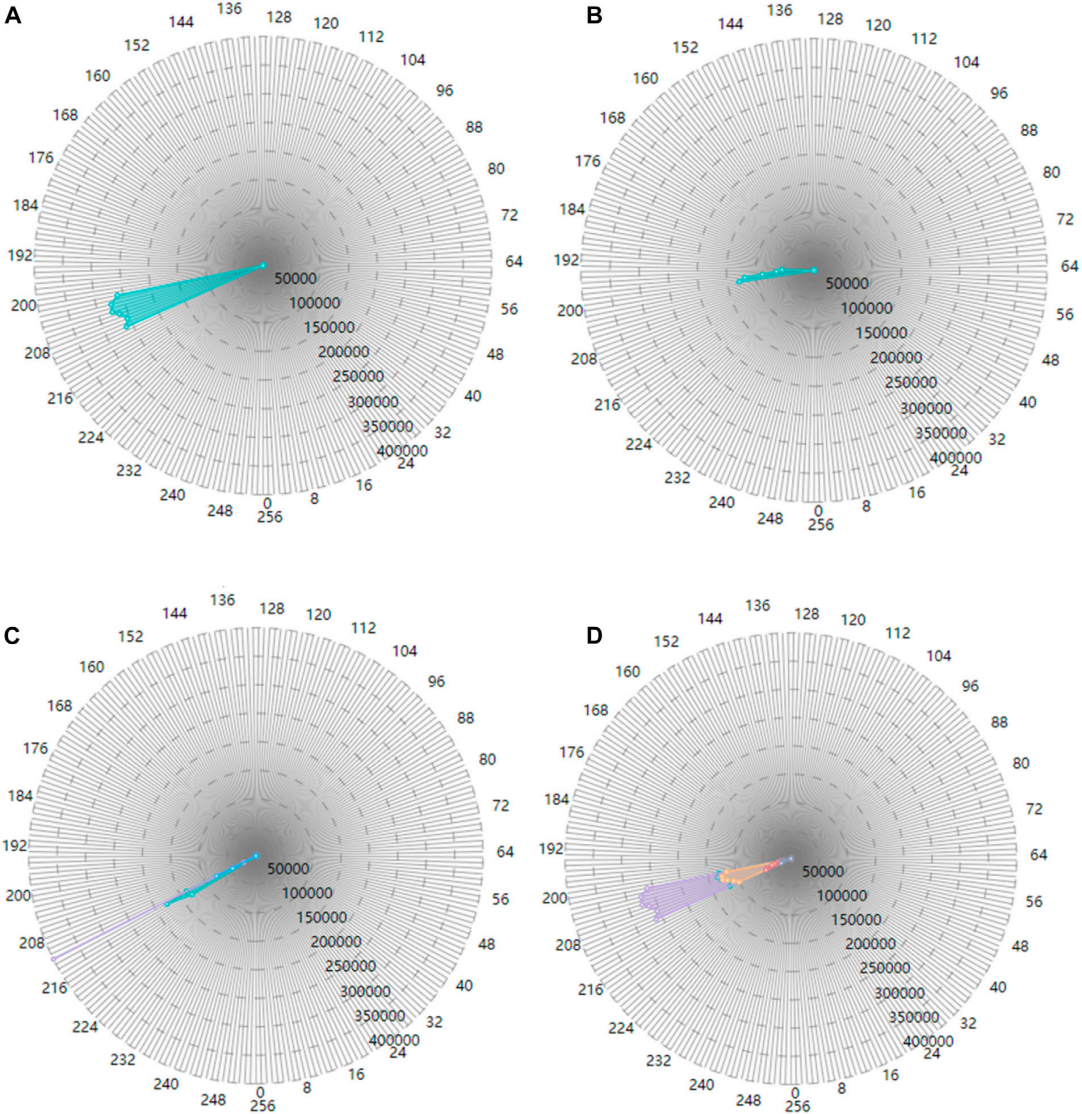
The correlation result displayed in the electrophysiology-myocyte module **(A)**. The result of the tissue value selected as 22 in the tissue-myocyte module and the action potential segment of 210 chosen in the tissue-electrophysiology module **(B)**. The result of the tissue value selected as 20 in the tissue-myocyte module and the action potential segment of 200 chosen in the tissue-electrophysiology module **(C)**. The result of the tissue value selected as 20, 22, and 25 with the action potential segment of 220 in the tissue-electrophysiology module. **(D)** The result of the issue value selected as 20, 21, 22, 23, 24, and 25 with the action potential segment of 210 in the tissue-electrophysiology module.

Figure 10A shows the number of myocytes with the action potential value within the selected segment of 210 in the left ventricle epicardium of value 22. Figure 10B shows the number of myocytes with the action potential value within the selected segment of 200 in the left ventricle endocardium of value 20. In Figure 10C, when the left ventricle endocardium, the left ventricle epicardium and epicardium of the right ventricle with value of 20, 22, and 25 respectively are simultaneously selected in the tissue-myocyte module, the number of myocytes in the three tissues with the action potential within the specific segment of 220 are displayed in the electrophysiology-myocyte module. While when the six tissues with values ranging from 20 to 25 are selected in the tissue-myocyte module and the specific action potential segment of 220 is selected in the tissue-electrophysiology module, the number of myocytes in these tissues are demonstrated in the electrophysiology-myocyte module, as shown in Figure 10D.

## 5 Conclusion

In this study, we propose a rendering framework to present the three-dimensional cardiac synergetic biological modality. Visual computing of cardiac synergetic modality is investigated and implemented to realistically present the three-dimensional cardiac structure and electrobiological activities. We build the biological modality histogram and designed the transfer function by interacting with the histogram. The local details of the heart are thus highlighted in the rendering result. In addition, cardiac physical-physiological correlation visualization is presented, and associations between physical and physiological modality are revealed. Our rendering framework also have a great advantage in cross-platform and rendering speed. In summary, this work provides an effective method for exploring the cardiac synergetic modality feature.

## Data Availability

The original contributions presented in the study are included in the article/Supplementary Material, further inquiries can be directed to the corresponding author.
