# Supplementary figures and images for: Cardiac biophysical detailed synergetic modality rendering and visible correlation
Source: Front Physiol. 2023 Apr 7;14:1086154. doi: 10.3389/fphys.2023.1086154 (PMC10119415; doi:10.3389/fphys.2023.1086154)

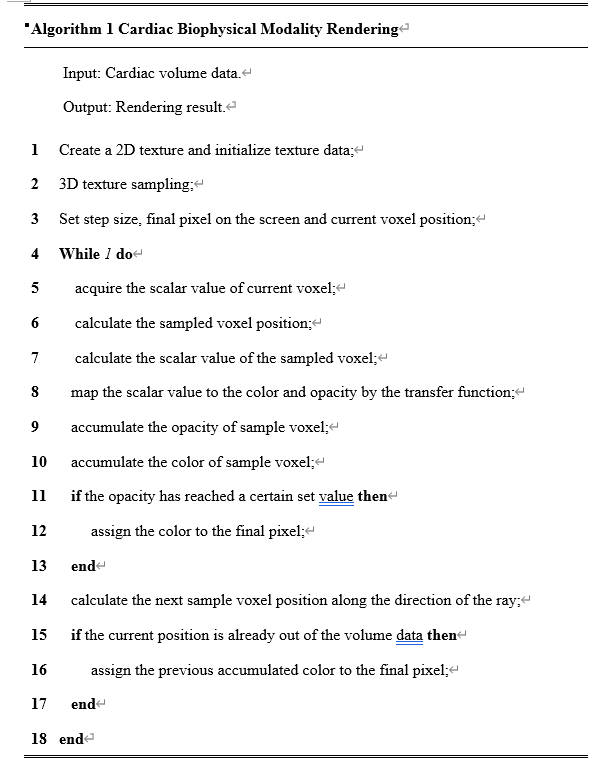

Supplement: Supplementary file 1 [file Image1.JPEG]
